# ^123^I-ADAM SPET imaging of serotonin transporter in patients with epilepsy and comorbid depression

**DOI:** 10.1186/1471-2377-13-204

**Published:** 2013-12-17

**Authors:** Maarika Liik, Malle Paris, Liina Vahter, Katrin Gross-Paju, Sulev Haldre

**Affiliations:** 1 Department of Neurology and Neurosurgery, University of Tartu, 8 L. Puusepa St., 51014 Tartu, Estonia; 2 Department of Nuclear Medicine, Radiology Centre, Diagnostics Division, North Estonia Medical Centre, 19 J. Sütiste St., 13419 Tallinn, Estonia; 3 Department of Neurology, West Tallinn Central Hospital, 68 Paldiski St., 10617 Tallinn, Estonia

**Keywords:** Epilepsy, Depression, Serotonin, Serotonin transporter, ^123^I-ADAM, SPET

## Abstract

**Background:**

Purpose of the study was to investigate alterations in midbrain serotonin transporter (SERT) binding in patients with epilepsy and symptoms of depression compared to patients with epilepsy with no symptoms of depression.

**Methods:**

We studied 12 patients with epilepsy (7 patients had focal and 5 had generalized epilepsy syndromes). The presence of self-reported symptoms of depression was assessed using Beck Depression Inventory (BDI) and the Emotional State Questionnaire (EST-Q). The binding potential of the SERT was assessed by performing brain single photon emission tomography (SPET) using the SERT radioligand 2-((2-((dimethylamino)methyl)phenyl)thio)-5-(123)iodophenylamine (^123^I-ADAM).

**Results:**

Seven patients had BDI and EST-Q subscale scores greater than 11 points, which was interpreted as the presence of symptoms of depression. We found that ^123^I-ADAM binding was not significantly different between patients with epilepsy with and without symptoms of depression. In addition, ^123^I-ADAM binding did not show a significant correlation to either BDI or EST-Q depression subscale scores and did not differ between patients with focal *vs.* generalized epilepsy.

**Conclusion:**

The results of our study failed to demonstrate alterations of SERT binding properties in patients with epilepsy with or without symptoms of depression.

## Background

The frequent coexistence of epilepsy and depression has encouraged researchers to explore potential shared mechanisms underlying these disorders. Depression occurs far more frequently in patients with epilepsy than in the general population, affecting approximately 30% of this patient population [1,2]. At the same time, the presence of a psychiatric disorder, such as depression, has been shown to reduce seizure threshold, and depression and attempted suicide themselves are risk factors for epilepsy [3-5]. These findings have led to the concept of a bidirectional relation between epilepsy and depression [6]. This issue is important, as the presence of depression is likely to be the most important factor influencing quality of life in patients with epilepsy [7,8], and can negatively impact both medical and surgical treatment outcomes [9-11].

The dysfunction of the brain serotonin (5-hydroxytryptamine or 5-HT) system has been suspected to be the common denominator for the shared pathogenic mechanisms of epilepsy and depression. Alterations in serotonergic signaling are associated with the pathogenesis of depression in patients with major depressive disorder - findings that are clinically supported by the effect of selective serotonin reuptake inhibitors (SSRIs) in the treatment of depression. Several neuroimaging studies using different positron emission tomography (PET) or single photon emission tomography (SPET) tracers for various components of serotonergic system in the brain have supported the involvement of 5-HT in major depressive disorder. These alterations include increased serotonin transporter (SERT) binding in the thalamus and limbic regions [12], or decreased brainstem and midbrain SERT binding [13-15], as well as reduced 5-HT_1A_ receptor binding potential in various limbic and neocortical regions and the raphe nuclei [16].

A growing body of evidence implicates the role of serotonin system in epilepsy. This includes studies in which the anticonvulsant effects of SSRIs have been confirmed, such as Favale et al. [17]. In this study, citalopram was administered to non-depressed patients with poorly controlled epilepsy who then experienced a marked drop in seizure frequency [17]. Previous neuroimaging works have used PET tracers for 5-HT_1A_ receptors to investigate the role of the serotonergic system in epilepsy and depression. These studies have concentrated on patients with both temporal lobe epilepsy (TLE) and depression and have shown reduced 5-HT_1A_ receptor binding potential in the ipsilateral temporal lobe as well as in thalamic regions, hippocampus, anterior insula, anterior cingulate, and the raphe nuclei in the depressed patients [18-20]. Thus, in TLE patients with depression, there appear to be alterations in the serotonergic system not only in the brain regions affected by epilepsy, but also more generally in ipsilateral and contralateral areas associated with regulation of emotion, these are changes that are similar to those described in patients with major depressive disorder alone.

2-((2-((dimethylamino)methyl)phenyl)thio)-5-(123)iodophenylamine (^123^I-ADAM) is a novel SPET tracer that has shown a high binding affinity for SERT as well as high selectivity for 5-HT transporter over those for norepinephrine and dopamine, and which has been proven to have excellent brain uptake in rats [21]. Subsequently, other studies demonstrated the feasibility of its use in human subjects [22-25]. Newberg et al. [26] used SPET to demonstrate alterations in SERT binding in patients with major depression; in this study, SERT binding was decreased in the midbrain region of patients with major depressive disorder and the degree of decrease correlated significantly with the severity of depressive symptoms [26]. These findings were generally corroborated by a later study by the same group using a larger sample size [27]. However, to our knowledge, no studies of SERT binding have been conducted in patients with epilepsy.

The aim of the current study was to investigate SERT binding in the midbrain of patients with epilepsy with symptoms of depression, and to determine differences in SERT binding compared to patients with epilepsy without symptoms of depression.

## Methods

### Subjects

This study was approved by the Research Ethics Committee of the University of Tartu. We studied 12 patients (7 men and 5 women; ages ranging from 21 to 55 years; mean age 36.3 ± 8.9 years) with epilepsy who were recruited from the outpatient clinic at the Department of Neurology and Neurosurgery in University of Tartu and West-Tallinn Central Hospital in Tallinn, Estonia. Patients were otherwise healthy, with no history of other neurological disorders except epilepsy, and did not use antidepressant medications prior to this study. All patients gave informed consent to participate in the study.

In the current study, all patients with symptoms of depression were consulted regarding their affective disorder, and treatment with antidepressant medications was offered following the ^123^I-ADAM SPET study.

### Assessment of symptoms of depression

Patients were screened for self-reported symptoms of depression using the Beck Depression Inventory (BDI) [28] and the Emotional State Questionnaire (EST-Q) [29]. Questionnaires were administered directly before the start of the ^123^I-ADAM SPET imaging session. A cut-off score of > 11 points was used to define the presence of symptoms of depression on the BDI. EST-Q is a self-report questionnaire for depression and anxiety that uses the rating of 33 items on a five-point frequency scale. This questionnaire has five subscales: depression, anxiety, agoraphobia–panic, fatigue, and insomnia. In the current study, a cut-off score of > 11 points was used to define the presence of symptoms of depression on the EST-Q depression subscale.

### Methods

We examined serotonin transporter (SERT) binding potential by performing brain SPET study with 2-((2-((dimethylamino)methyl)phenyl)thio)-5-(123)iodophenylamine (^123^I-ADAM). The subjects received a dose of 185 MBq ^123^I-ADAM (MAP Medical Finland) intravenously. To block the thyroid gland, potassium perchlorate (KClO4; 800 mg) was given orally at least 20 min prior to the injection of ^123^I-ADAM. Brain SPET studies were acquired 4 hours after the injection of ^123^I-ADAM.

SPET studies were performed using a SPET/CT INFINIA Hawkeye 4 (GE Healthcare) dual head gamma camera with low energy high-resolution collimators (Lehr collimators). The energy window was centered on 159 keV (+/-10%). SPET scans were acquired in a step and shoot mode with total angular range of 360 degrees thereby arc per detector being 180 degrees. View angle 3 degrees, 120 views, 30 sec per projection. Acquisition time was 30 min. Matrix size was 128 × 128, with a zoom of 1.0.

Data were reconstructed using the Xeleris Functional Imaging Workstation software (GE Healthcare). Transverse slices were reconstructed parallel to the canthomental plane. SPET data were reconstructed using a Butterworth filter (critical frequency 0.4, power 6), followed by the Chang attenuation correction (threshold 5, coefficient 0.11). SPET and MRI data were automatically coregistered using MPI Tool software (ATV Inc., Kerpen, Germany). To measure the individual SERT occupancy, irregular regions of interest (ROIs) were manually drawn over the midbrain and over the cerebellum as the reference area. The ^123^I-ADAM binding was assessed using MRI-guided ROIs in the midbrain and cerebellum. ROIs were placed on transaxial MRI slices over the midbrain and cerebellum and then transferred onto corresponding SPET slices. In addition, radio-uptake and the specific uptake ratios (SURs) of midbrain were assessed. As a measure of brain SERT availability, the ratio of specific-to-nonspecific ^123^I-ADAM binding for the midbrain compared to the cerebellum were calculated in mean counts/pixel using the following equation: SUR = specific binding/nonspecific binding = target- cerebellum/cerebellum.

### Statistical analysis

Statistical analysis was performed with STATISTICA 8.0. Student’s *t*-tests were used to compare variables between the two groups of patients (with symptoms of depression *vs*. without symptoms of depression). A correlation analysis was used to assess the relationship between depression scale scores, demographic, and clinical characteristics, and SERT binding potential.

## Results

A summary of patient characteristics, including their demographic and clinical characteristics is included in Table 1. Seven patients had focal epilepsy; six of these had had long-term EEG monitoring performed in order to define focus localization. Of these seven patients, six patients had TLE (two with right sided and four with left sided TLE) and one patient had probable frontal lobe epilepsy (FLE; lateralization to the right side). Five patients had generalized epilepsy syndrome. MRI scans for the generalized epilepsy patients were all normal. On the MRI, three patients with focal epilepsy presented with mesial temporal sclerosis, one with hippocampal atrophy, and one with cysts in temporomesial structures. Epilepsy could be considered treatment resistant in the majority of the patients, eight of whom were on polytherapy with antiepileptic drugs (AEDs). The patients with focal epilepsy, as a group, were comparable to the patients with generalized epilepsy regarding age, age at epilepsy onset, epilepsy duration, use of AEDs, as well as their mean BDI and EST-Q questionnaire scores.

**Table 1:** **Demographic and clinical characteristics, presence of symptoms of depression and midbrain **^**123**^**I-ADAM binding potential to SERT of individual patients**

**Age (years)**	**Sex**	**Age at onset (years)**	**Duration of epilepsy (years)**	**Frequency of seizures**	**Syndrome**	**Localization**	**Interictal EEG**	**LTM**	**MRI**	**AEDs (daily dose, mg)**	**Symptoms of depression**	^**123**^**I-ADAM binding to SERT**
32	M	17	15	2-5/mo	Generalized	N/A	Generalized spike-wave activity	Not performed	Normal	LEV (2000)	No	1.3
										VPA (1800)		
										LTG (75)		
24	F	5	19	1/mo	Generalized	N/A	Generalized spike-wave activity	Not performed	Normal	VPA (1500)	No	1.39
										OXC (1200)		
36	M	21	15	1/year	Generalized	N/A	Negative	Not performed	Normal	VPA (600)	No	0.87
21	F	15	6	3/week	Generalized	N/A	Generalized spike-wave activity	Generalized tonic.clonic seizures	Normal	LTG (400)	Yes	1.36
										OXC (600)		
55	M	23	32	1/mo	Generalized	N/A	Generalized spike-wave activity	Not performed	Normal	VPA (2000)	Yes	1.16
										LTG (150)		
39	M	27	12	2/year	Focal	Left temporal	Left temporal interictal discharges	Left temporal ictal activity	Small cysts in left temporomesial structures, hippocampus and amygdala	LTG (250)	No	1.42
40	F	24	16	4-5/mo	Focal	Left temporomesial	Left temporal interictal discharges	Left temporal ictal activity	Colloidal cyst in left frontotemporal regions	PB (50)	No	1.1
										LTG (200)		
38	M	22	16	1/mo	Focal	Left temporal	Negative	Not performed	Hippocampal atrophy on the left side	OXC (1800)	Yes	0.98
										VPA (900)		
42	M	4	38	4-6/mo	Focal	Right temporomesial	Right temporal interictal discharges	Right temporal ictal activity	Mesial temporal sclerosis on the right side	PHT (375)	Yes	1.28
42	F	14	28	2/mo	Focal	Right frontal	Negative	Right frontal ictal activity	2 small hyperintensive lesions in the right parietal lobe	OXC (1800)	Yes	1.46
34	F	7	27	4-5/week	Focal	Left temporomesial	Left temporal interictal discharges	Left temporal ictal activity	Mesial temporal sclerosis on the left side	VPA (1000)	Yes	1.32
										TPM (100)		
32	M	2	30	3/week	Focal	Right temporomesial	Right temporal interictal discharges	Right temporal ictal activity	Mesial temporal sclerosis on the right side	OXC (1800)	Yes	1.26
										TPM (400)		

EEG – electroencephalography; LTM – long-term monitoring; MRI – magnetic resonance imaging; AEDs – antiepileptic drugs; LEV – levetiracetam; VPA – valproate; LTG – lamotrigin; OXC – oxcarbazepine; PB – phenobarbital; PHT – phenytoin; TPM – topiramate; ^123^I-ADAM – 2-((2-((dimethylamino)methyl)phenyl)thio)-5-(123)iodophenylamine; SERT – serotonin transporter.

Seven patients had BDI and EST-Q depression subscale scores greater than 11 points, which was interpreted as the presence of symptoms of depression. The mean BDI and EST-Q depression subscale scores for the whole patient group were 11.5 ± 6 and 14 ± 4.3, respectively. The maximal BDI score was 20; in all patients with symptoms of depression, the BDI score was in the range of mild to moderate severity.

In the current study, patients with symptoms of depression, as a group, did not significantly differ from patients without symptoms of depression in either demographic or clinical variables (Table 2). A comparison of these patient groups indicated that patients with symptoms of depression showed a trend towards a longer duration of epilepsy, which did not reach statistical significance.

**Table 2:** **Comparison of clinical characteristics, depression questionnaire results and midbrain SERT binding affinity on **^**123**^**I-ADAM between groups of patients with symptoms of depression (BDI and EST-Q score > 11 points) and without symptoms of depression**

	**Symptoms of depression (n=7)**	**No symptoms of depression (n=5)**	***P*-value**
Age	37.7 (± 10.5)	34.2 (±6.5)	NS
Age at onset	12.4 (±8.4)	18.8 (±8.6)	NS
Duration of epilepsy	25.3 (±10.8)	15.4 (±2.5)	NS
BDI	15.3 (±3.8)	6.0 (±1.4)	0.004
EST-Q	15.8 (±2.6)	7.0 (±1.0)	0.04
^123^I-ADAM binding to SERT	1.26 (±0.2)	1.216 (±0.2)	NS

NS – not significant; BDI – Beck Depression Inventory; EST-Q - Emotional State Questionnaire; ^123^I-ADAM – 2-((2-((dimethylamino)methyl)phenyl)thio)-5-(123)iodophenylamine; SERT – serotonin transporter.

Using SPET, we observed that ^123^I-ADAM binding to SERT did not differ significantly between the patients with epilepsy who had symptoms of depression *vs*. those without. In addition, SERT binding potential of ^123^I-ADAM did not show any statistically significant correlation with either the BDI or the EST-Q depression subscale scores. SERT binding potential was also not correlated with any demographic or clinical characteristics, including age, duration of epilepsy, or age at disease onset. We also observed that the SERT binding potential did not differ between patients with focal *vs*. generalized epilepsy.

One patient with moderate symptoms of depression on BDI committed suicide shortly following the ^123^I-ADAM imaging study.

## Discussion

In the current study, we sought to study SERT binding properties in the midbrain region in patients with epilepsy, and to determine whether SERT binding differed between depressed vs. non-depressed patients with epilepsy. Our results did not indicate any difference in SERT binding potential between these patient groups.

There could be several reasons for these negative results. Previous work with PET and SPET tracers for SERT in depressed patients has shown some conflicting results. The majority of reports show increased SERT binding in the thalamus and limbic regions of depressed patients compared to controls [30], but others have shown decreased SERT binding potential in the amygdala and midbrain of depressed patients [13-15]. Studies using ^123^I-ADAM SPET to measure SERT binding in major depressive disorder have also indicated decreased SERT binding in the midbrain, medial temporal lobe, and basal ganglia of depressed patients compared to controls [26,27]. At the same time, however, reports showing no differences in midbrain SERT availability for ^123^I-ADAM in patients with depression compared to healthy controls have also been published [31,32]. It has been hypothesized that, in case of major depressive disorder, the SERT binding potential is elevated, but that in major depressive disorder with comorbid psychiatric illnesses, regional SERT binding could be decreased [12]. Taking this into account, and considering the fact that there are no SERT binding studies done in patients with epilepsy, it could be difficult to predict the directionality of alterations in SERT binding in patients with epilepsy and comorbid depression. Addressing this hypothesis more fully would likely require a study with a larger sample size.

Another contributing factor to these negative results may be the genetic variability that has been shown for SERT expression in the human brain. For example, individuals with polymorphisms in the promoter (5-HTTLPR) of the *SLC6A4* gene, which encodes the SERT protein, exhibit differences in SERT binding properties in neuroimaging studies [33-35]. Genetic studies have shown that there may even be an association between the presence of the combined 5-HTTLPR and 5-HTTVNTR genotype, which results in less efficient transcription of SERT, and the presence of TLE [36]. Genetic variability of SERT expression may influence the development of affective disorders, it would likely affect SERT imaging studies, and could even be related to epileptogenesis. Unfortunately, in the current study, we did not genotype our patients for polymorphisms in *SLC6A4*.

There are several limitations to the current study. Perhaps the most important, and the one likely to be largely responsible for our negative results, is the relatively small study sample size. The statistical power of the comparison of SERT binding in groups of patients with and without depression was 0.755. We calculated that increasing the power to 0.8 under the same conditions would need 40 subjects, which considering the nature and cost of SPET imaging, would be unachievable.

Since in all patients with symptoms of depression, the BDI score was in the range of mild to moderate severity, it could also be considered as a weakness contributing to the negative results of our study.

The heterogeneity of our study group, regarding the clinical characteristics of epilepsy, could also have led to the observed lack of differences in SERT binding properties. The characteristics of depression, depression-related treatment outcomes, and serotonergic system involvement, based on 5-HT_1A_ receptor imaging studies, are all well-documented in cases of TLE. Little is known about the same aspects in case of focal extra-temporal epilepsies, and even less about generalized epilepsy syndromes. Although some findings have indicated that depression could be specifically related to TLE and mesial temporal sclerosis [37], this has not been confirmed by other reports [38]. It has been shown that the prevalence of depression is similar between patients with TLE and FLE. There are no reports of focal *vs*. generalized epilepsy in terms of the prevalence of depression. One work did assess symptoms of anxiety, and found that patients with FLE have much higher anxiety scores than patients with generalized epilepsy [39]. In our study, groups of patients with focal *vs.* generalized epilepsy were comparable in terms of presence of depressive symptoms.

These previous findings seem to indicate that the bidirectional relationship between epilepsy and depression is not specific to TLE. It has been shown that preoperative depressive symptoms predict postoperative seizure outcome in both TLE and FLE [11]. Therefore, common pathogenic mechanisms may be involved in the etiology of depression comorbid with different epilepsy syndromes, and we would expect that this should be demonstrable in patients having different clinical characteristics of epilepsy, such as those included in the current study. Our findings support the notion that depression and the involvement of the serotonergic system in various epilepsy syndromes requires a deeper exploration with further studies.

## Conclusions

The results of our study failed to demonstrate alterations of SERT binding potential in patients with epilepsy with symptoms of depression compared to patients with epilepsy without symptoms of depression. Further studies are needed to clarify the role of SERT and, more generally, the serotonergic system in the common pathogenesis of epilepsy and depression.

## Abbreviations

123I-ADAM: 2-((2-((dimethylamino)methyl)phenyl)thio)-5-(123)iodophenylamine; SPET: Single photon emission tomography; SERT: Serotonin transporter; BDI: Beck depression inventory; EST-Q: Emotional state questionnaire; 5-HT: Serotonin; SSRIs: Selective serotonin reuptake inhibitors; PET: Positron emission tomography; TLE: Temporal lobe epilepsy; ROIs: Regions of interest; MRI: Magnetic resonance imaging; FLE: Frontal lobe epilepsy; AEDs: Antiepileptic drugs

## Competing interests

The authors declare that they have no competing interests.

## Authors’ contributions

ML participated in the conception and design of the study, acquisition and analysis of data, and drafted the manuscript; MP participated in the design of the study, acquisition of SPET data, and revised the manuscript critically; LV participated in the conception of the study, acquisition of data, and revised the manuscript critically; KGP participated in the conception of the study, acquisition of data, and revised the manuscript critically; SH participated in the conception and design of the study, coordinated the study, and revised the manuscript critically. All listed authors have read the manuscript and have given their final approval of the version to be published.

## Pre-publication history

The pre-publication history for this paper can be accessed here:

http://www.biomedcentral.com/1471-2377/13/204/prepub
